# Comprehensive Comparison of Amnion Stromal Cells and Chorion Stromal Cells by RNA-Seq

**DOI:** 10.3390/ijms22041901

**Published:** 2021-02-14

**Authors:** Brielle Jones, Chaoyang Li, Min Sung Park, Anne Lerch, Vimal Jacob, Nicholas Johnson, Jin-Qiang Kuang, Sandeep Dhall, Malathi Sathyamoorthy

**Affiliations:** Smith and Nephew Inc., 7015 Albert Einstein Drive, Columbia, MD 21046, USA; briellejones@gmail.com (B.J.); minsung.park@smith-nephew.com (M.S.P.); anne.lerch@smith-nephew.com (A.L.); vimal.jacob@smith-nephew.com (V.J.); nicholas.johnson@smith-nephew.com (N.J.); jinqiang.kuang@smith-nephew.com (J.-Q.K.)

**Keywords:** amniotic stromal cells, chorionic stromal cells, angiogenesis, anti-inflammation, RNA-seq, placenta

## Abstract

Mesenchymal stromal cells derived from the fetal placenta, composed of an amnion membrane, chorion membrane, and umbilical cord, have emerged as promising sources for regenerative medicine. Here, we used next-generation sequencing technology to comprehensively compare amniotic stromal cells (ASCs) with chorionic stromal cells (CSCs) at the molecular and signaling levels. Principal component analysis showed a clear dichotomy of gene expression profiles between ASCs and CSCs. Unsupervised hierarchical clustering confirmed that the biological repeats of ASCs and CSCs were able to respectively group together. Supervised analysis identified differentially expressed genes, such as *LMO3*, *HOXA11*, and *HOXA13*, and differentially expressed isoforms, such as *CXCL6* and *HGF*. Gene Ontology (GO) analysis showed that the GO terms of the extracellular matrix, angiogenesis, and cell adhesion were significantly enriched in CSCs. We further explored the factors associated with inflammation and angiogenesis using a multiplex assay. In comparison with ASCs, CSCs secreted higher levels of angiogenic factors, including angiogenin, VEGFA, HGF, and bFGF. The results of a tube formation assay proved that CSCs exhibited a strong angiogenic function. However, ASCs secreted two-fold more of an anti-inflammatory factor, TSG-6, than CSCs. In conclusion, our study demonstrated the differential gene expression patterns between ASCs and CSCs. CSCs have superior angiogenic potential, whereas ASCs exhibit increased anti-inflammatory properties.

## 1. Introduction

The placenta, which functions as a fetomaternal organ, is composed of both fetal tissues and maternal tissues by structure. The amnion membrane, chorion membrane, and umbilical cord are the fetal parts of the placenta. These tissues have been shown to support restoration of several tissue types, including bone, tendon, and skin tissue [1,2,3]. The observed plethora of biological effects are due to the activation of epithelialization and neovascularization, suppression of inflammation and scarring, pain reduction, and inherent antimicrobial properties [4,5,6,7,8,9,10]. The mesenchymal stromal cells (MSCs), rich extracellular matrix, key cytokines, and growth factors in the tissues contribute to these functional properties. There is wide acceptance that the extracellular matrix, cytokines, and growth factors are mainly derived from the secretion of the MSCs within the tissues. Therefore, the MSCs residing in the tissues at least partially dominate the functional properties of the amnion membrane, chorion membrane, and umbilical cord.

MSCs have the potential for use in regenerative medicine and have been investigated in a number of clinical trials for currently untreatable diseases, such as myocardial infarction, stroke, graft-versus-host disease, bone and cartilage defects, and autoimmune diseases [11,12,13,14]. Since bone-marrow-derived MSCs (BM-MSCs) were first reported, a number of studies have shown similar cell types in a wide range of tissues, including the placental fetal membrane, umbilical cord, adipose tissue, dental tissue, skin, hair follicles, and tonsils. Recently, comparative studies showed that MSCs derived from fetal placental tissues are more prone to differentiating into osteoblasts than BM-MSCs are. BM-MSCs and adipose-tissue-derived MSCs (AD-MSCs) represent the optimal stem cell source for tissue engineering and regenerative medicine [15,16]. However, as sources of MSCs, placental tissues present several advantages for clinical usage compared with other MSC sources [17,18,19,20,21,22]. These include the absence of ethical concerns, their uncomplicated harvest protocol, avoidance any invasive procedure on the donors, and, importantly, the young cellular age of the donors. In addition, the factors secreted by placental MSCs are more adept at angiogenesis, cell proliferation, differentiation, cell survival, immunomodulation, and collagen degradation than those of AD-MSCs [23,24].

MSCs can be isolated from all layers of a full-term placenta, including the amniotic membrane, chorionic membrane, chorionic villi, decidua, and umbilical cord [25,26,27,28]. Comparing MSCs from different parts of the placenta will facilitate the selection of appropriate MSCs for clinical applications. Several studies have isolated and investigated the characteristics of these cells after in vitro culture. However, controversial results between different research groups have rendered the outcomes inconclusive. Soncini et al. (2007) showed that both amnion MSCs and chorion MSCs are comparable with regard to multi-lineage differentiation [29]. However, Choi et al. (2017) showed that chorion MSCs have higher multi-lineage differentiation potential, and both chorion MSCs and chorionic villi MSCs tend to have higher proliferative potentials than amnion MSCs [30]. Yamahara et al. (2014) showed that amnion and chorion MSCs secrete differential soluble factors and showed differences in their angiogenic and immune-suppressive functions [31]. However, Wu et al. (2018) showed that MSCs from the umbilical cord and amnion exhibited superior growth potential and higher anti-inflammatory properties than chorion MSCs [32], whereas, chorion MSCs displayed potential pro-angiogenic activity due to the higher secretion of HGF and VCAM-1 [32]. Moreover, Huang et al. (2019) showed that chorion MSCs demonstrated significantly stronger proliferation ability and immunomodulatory properties in comparison with MSCs derived from other layers of the placenta [26]. Kim et al. (2011) concluded that chorion MSCs, rather than chorionic villi MSCs, are useful sources of cells for appropriate clinical applications in the treatment of various degenerative diseases [33]. Kannaiyan et al. (2018) showed that MSCs from the amnion and chorionic villi were capable of differentiating into cardiac progenitor MSCs and had comparable angiogenic capacity [34]. Taken together, further investigation is needed to better understand the functional properties of MSCs derived from different sources of placental tissues, and this will potentially impact future clinical applications. This may be achieved by identifying the molecular pathways and cytokine profiles of these MSCs.

Among the MSCs derived from different layers of the placental tissue, amnion and chorion MSCs have advantages as cell sources, as a large quantity of cells can be obtained because of their size and the minimal contamination with the original maternal cells. To comprehensively compare amnion and chorion MSCs at the molecular and signaling levels, in the present study, we used next-generation sequencing technology to explore the differences between freshly isolated amnion MSCs and chorion MSCs at the molecular level. The morphology and immunophenotype of the isolated MSCs were analyzed. The cytokine secretion profiles were quantitatively evaluated with an enzyme-linked immunosorbent assay and multiplex cytokine arrays. We further investigated the anti-inflammatory and pro-angiogenic functions of the MSCs derived from both the amnion and chorion. Our data suggest that amnion MSCs exhibit strong anti-inflammatory functions, whereas chorion MSCs show increased pro-angiogenic signaling and increased secretion of angiogenic factors.

## 2. Results

### 2.1. Characterization of ASCs and CSCs

**Figure 1:**
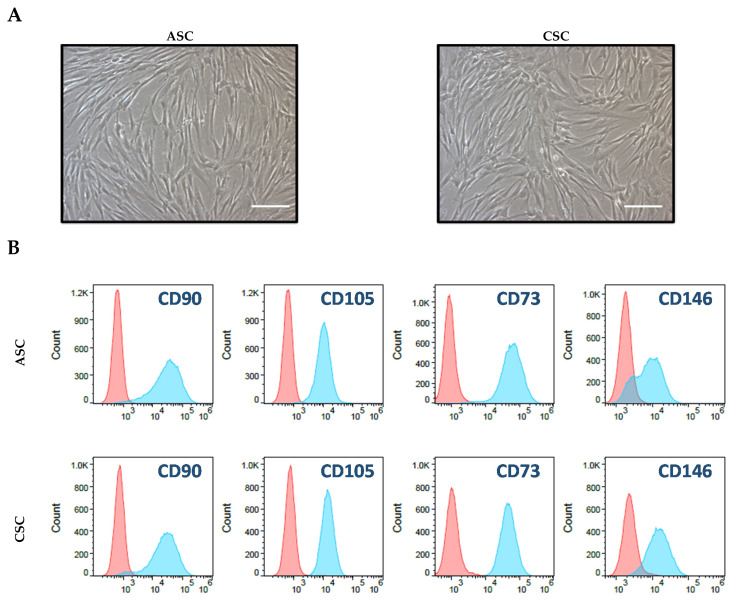

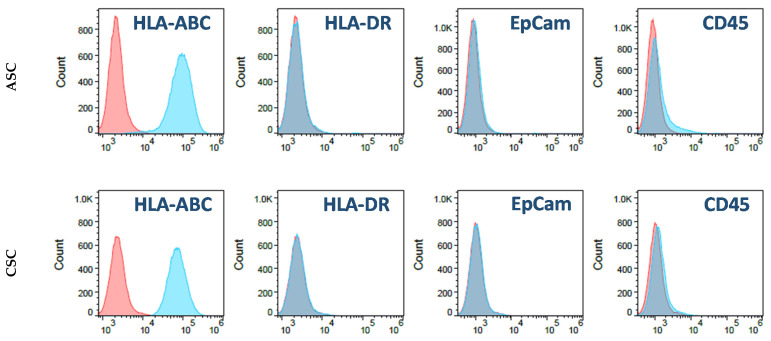
Characterization of amniotic stromal cells (ASCs) and chorionic stromal cells (CSCs). (**A**) Morphological appearance of ASCs and CSCs. A total of 5 × 10^5^ isolated primary ASCs and CSCs were plated in T175 tissue culture plates, and pictures were taken on day 10 under a regular microscope. Scale bar = 100 μM. (**B**) Flow cytometry analysis of cultured ASCs and CSCs for the indicated antibodies (blue) or their corresponding isotype control antibodies (red).

To characterize the isolated amniotic stromal cells (ASCs) and chorionic stromal cells (CSCs), we first cultured these cells in a low-glucose DMEM medium containing 5% fetal bovine serum (FBS) in tissue-culture-treated flasks. We found that both ASCs and CSCs attacheded to the tissue-culture flasks and showed a fibroblast-like morphology under a microscope (Figure 1A). We then tested cell-surface markers with flow cytometry to verify that these cells were truly MSCs. We found that these cells expressed high levels of stromal cell markers, such as CD90, CD105, CD73, CD146, and HLA-ABC; they showed HLA-DR and EpCam and were CD45 negative (Figure 1B). CD90, CD105, CD73, and CD146 are common mesenchymal stromal cell markers. These cells expressed high levels of HLA-ABC but not HLA-DR, which revealed that they were undifferentiated MSCs. No cells were EpCam or CD45 positive, indicating that there was no epithelial cell or immune cell contamination, respectively. Overall, we found that both ASCs and CSCs exhibited morphological characteristics of MSCs and expressed common MSC markers. Unfortunately, we did not see differences between ASCs and CSCs regarding their morphology and cell surface marker expression.

### 2.2. Principal Component Analysis of the ASCs and CSCs

To better understand the differences in global gene expression levels and to identify new tissue-specific and tissue-enriched regulators, we performed RNA-seq-based expression profiling of the MSCs using three primary ASCs and four primary CSCs isolated from human placental tissues. Toward this end, we isolated total RNA from ASCs and CSCs. Biological repeats of these RNA samples were sequenced at high depth using an Illumina NexSeq500. The sequence reads were mapped to the reference genome sequence of Homo Sapiens (GRCh37) using Tophat (v2) and Bowtie2. Subsequently, between-sample normalization was performed using the median normalization method, and the number of fragments per kilobase of transcript per million mapped fragments (FPKM) was calculated using Cuffdiff. The calculated FPKM will give us measurements of the relative expression of genes within and between biological samples.

**Figure 2:**
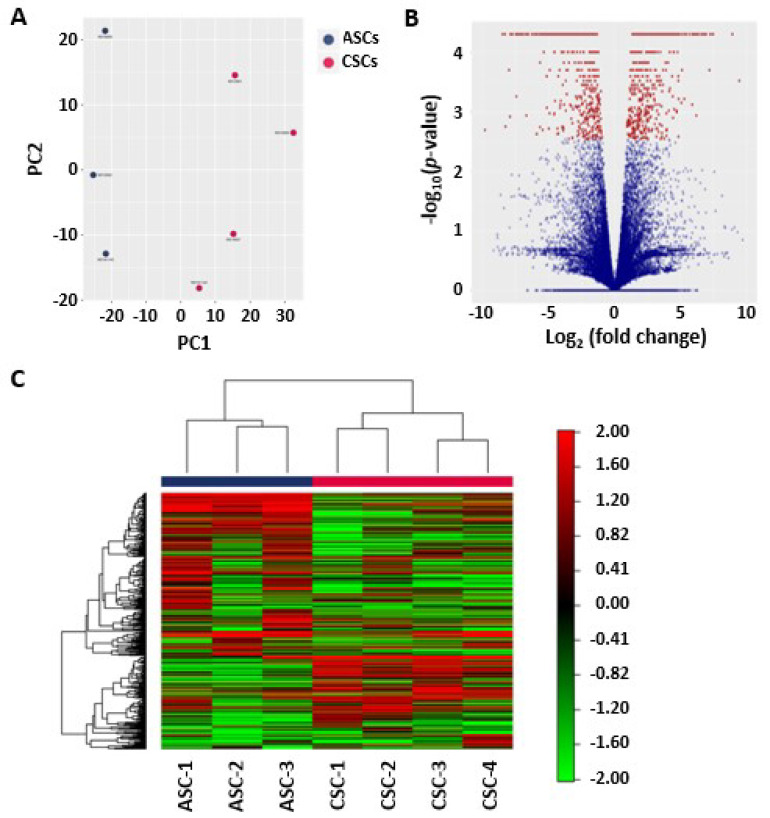
Differential expression patterns between ASCs and CSCs. (**A**) Principal component analysis (PCA) plot for ASCs and CSCs. The PCA was performed on all samples that passed the quality control using the 500 genes that had the largest coefficients of variation based on the fragments per kilobase of transcript per million mapped fragments (FPKM) counts. Each circle represents a sample. (**B**) Volcano plot showing the relationship between the raw *p*-values and the log2-fold change in normalized expression (FPKM) between ASCs and CSCs. (**C**) Heat map and unsupervised hierarchical clustering by sample and gene were performed on the listed samples using the 500 genes that had the largest coefficients of variation based on the FPKM counts. The data are based on samples from the ASC and CSC groups.

In order to better analyze and appreciate the overall expression patterns between ASCs and CSCs, we utilized principal component analysis (PCA), a statistical technique that reduces and summarizes large datasets while illustrating relationships between samples based on co-variance of the data being examined. The PCA was performed on all samples using the 500 genes that had the largest coefficients of variation based on the FPKM counts. Using PCA, we found that principal component 1 (PC1) and principal component 2 (PC2) accounted for approximately 90% of all variations of the original data. To further explore and better depict the major sources of variation, all samples were plotted in a two-dimensional space consisting of PC1 and PC2. Interestingly, as demonstrated in Figure 2A, samples of the ASC group and CSC group segregated from each other, demonstrating the differential gene expression between these two groups. However, biological replicates did not cluster tightly together, suggesting variations among different donors. Taken together, these analyses provided the first hint of a clear dichotomy of gene expression profiles between ASCs and CSCs.

### 2.3. Heat Map and Unsupervised Hierarchical Clustering

To perform a quick visual identification of the genes displaying large-magnitude changes that are also statistically significant, we generated a volcano plot (Figure 2B). The plot was constructed by plotting the *p*-value on the y-axis and the expression fold change between ASCs and CSCs on the x-axis. We identified 653 highly statistically significant genes, among which 307 genes were strongly up-regulated in ASCs and 346 were strongly up-regulated in CSCs (Figure 2B). Clustering of RNA-seq data can be used to identify patterns of gene expression by grouping genes based on their distance in an unsupervised manner. To visualize relationships between groups of genes, using the 500 genes that had the largest coefficients of variation based on the FPKM counts, we generated a heat map diagram through unsupervised hierarchical clustering (Figure 2C). We found, as expected, that biological repeats of ASCs and CSCs were respectively grouped together.

### 2.4. Differentially Expressed Genes and Novel Isoforms

We performed supervised analysis and generated a list of the differentially expressed genes. The top 10 most significantly differentially expressed genes are shown in Table 1. These genes were *NAALL*, *LMO3*, *UTY*, *BIRC7*, *APOD*, *HOXA13*, *MKRN4P*, *HOXA 11*, *RGPD1*, and *NR2F1*. Among these genes, *NAALL*, *BIRC7*, *APOD*, *HOXA13*, *MKRN4P*, *HOXA 11*, *RGPD1*, and *NR2F1* were highly expressed in CSCs; *LMO3* and *UTY* were highly expressed in ASCs. We also identified the 10 most significantly differentially expressed isoforms (Table 2), including both known and novel isoforms (Table 3). These isoforms included *CXCL6, FOXF1-AS1, HGF, MAEL, NPTX1, HGF, ATP2A3, DPT, POSTN*, and a novel isoform (Isoform ID: XLOC_040015). Among these novel isoforms, *HGF, MAEL, NPTX1, ATP2A3,* and *DPT* were highly expressed in CSCs, and *CXCL6, FOXF1-AS1, POSTN,* and the novel isoform (Isoform ID: XLOC_040015) were highly expressed in ASCs. The genes located closest to the differentially expressed novel isoforms were *HGF*, *RP11*, *MICAL2*, *KRT7*, *PERP*, *ACRC*, *PLBD1*, *IL1B*, *MEST*, and *CSF1*. Alternative splicing is an important posttranscriptional process that enables a single gene to produce multiple distinct transcripts, namely isoforms [35]. These isoforms carry different biological properties that are different in catalytic ability, subcellular localization, or protein interaction.

### 2.5. GO Enrichment Analysis

**Figure 3:**
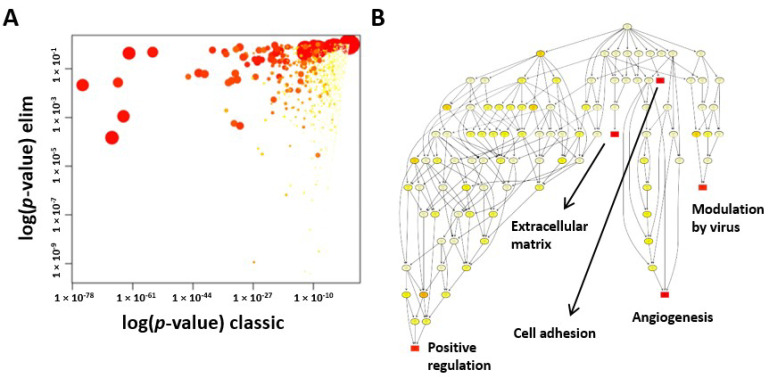
Gene Ontology (GO) analysis of ASCs and CSCs. (**A**) Scatter plot of the significantly enriched GO terms associated with genes that were differentially expressed between the ASCs and CSCs. The plot shows a comparison of the results obtained by the two statistical tests used. Values along diagonal were consistent between both methods. Values in the bottom left of the plot correspond to the terms with the most reliable estimates from both methods. The size of a dot is proportional to the number of genes mapping to that GO term, and the color represents the number of significantly differentially expressed transcripts corresponding to that term, with dark red representing more terms and yellow representing fewer. (**B**) The GO network generated for the enriched GO terms (biological process) associated with genes that were differentially expressed between the ASCs and CSCs. The nodes are colored from red to yellow, with the node with the strongest support colored red and nodes with no significant enrichments colored yellow. The five nodes with the strongest support are marked with rectangular nodes.

We used GO enrichment analysis to investigate whether specific GO terms were more likely to be associated with the differentially expressed transcripts. Two different statistical tests were used and compared. Firstly, a standard Fisher test was used to investigate enrichment of terms between the two groups. Secondly, the “Elim” method, which takes a more conservative approach by incorporating the topology of the GO network to compensate for local dependencies between GO terms and can mask the significant GO terms, was used. Comparisons of the predictions from these two methods were able to highlight truly relevant GO terms (Figure 3A). We first performed a comparison of the results for the GO terms associated with the significantly differentially expressed transcripts that were identified between the two groups. We generated a scatter plot for significantly enriched GO terms associated with genes that were differentially expressed between the ASCs and CSCs. To illustrate how the different GO terms were linked, we created a GO network (Figure 3B). The top 10 most significant GO terms are given in Table 4. Among them, the most interesting three GO terms are extracellular matrix organization, angiogenesis, and cell adhesion, which are essential for regenerative functions. In particular, the angiogenic function of CSCs drew our attention.

### 2.6. CSCs Secrete Significant Amounts of Angiogenic and Inflammatory Factors

To further characterize the CSCs, we cultured the CSCs in vitro and analyzed the levels of secreted factors and cell lysates involved in angiogenesis and inflammation. We quantitated each individual factor using a multiplex assay kit. As shown in Table 5, the CSCs secreted high levels of angiogenic factors, such as angiogenin, VEGFA, HGF, and FGF-2. However, the levels of these factors in ASCs remained undetectable. Both ASCs and CSCs also secreted variable levels of inflammatory factors, as shown in Table 5.

### 2.7. CSCs Exhibit Increased Angiogenic Function

**Figure 4:**
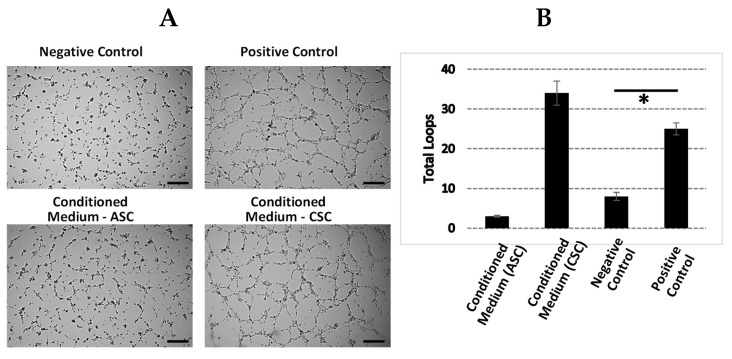

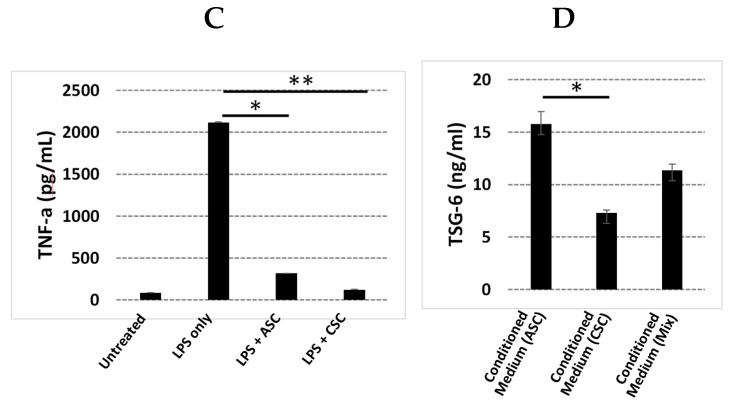
Angiogenic and anti-inflammatory functions of ASCs and CSCs. (**A**) Factors secreted by CSCs induced angiogenesis. The conditioned media collected from in vitro cultured ASCs and CSCs were used for the tube formation assay. Negative control: no serum medium; positive control: medium containing 5% fetal bovine serum (FBS). Representative images of tube formation are shown. Scale bar = 500 µM. (**B**) Quantitative loops were counted, and the averages of three experimental repeats are shown. (**C**) ASCs and CSCs showed an anti-inflammatory function. THP-1 cells were treated with lipopolysaccharide (LPS) in the presence or absence of ASCs and CSCs for 48 h. The collected medium was used to detect the level of TNF-a with ELISA. Non-LPS-treated samples were used as a negative control. (**D**) ASCs secreted higher levels of TSG-6 than CSCs. The conditioned media collected from the cultured ASCs and CSCs were used for TSG-6 analysis with ELISA. Error bar = standard deviation. * *p* < 0.05; ** *p* < 0.001.

We next evaluated whether the angiogenic function in CSCs enriched through GO analysis was associated with improved angiogenic function. We performed a tube formation assay by using a conditioned medium collected from cultured ASCs and CSCs (Figure 4A). We found that the conditioned medium from the CSCs significantly increased the number of loops (Figure 4B), suggesting its enhanced angiogenic function in vitro. However, the conditioned medium from the ASCs only slightly increased the number of tube formations. In addition, we also collected the amnion and chorion tissues and analyzed the levels of key factors involved in angiogenesis and anti-inflammation. The results showed that, in comparison with amnion tissues, chorion tissues harbored higher levels of angiopoitein-2, SDF-1a, IGFBP-1, PDGF-AA, PDGF-BB, VEGF-A, and VEGF-D (Table 6). These results demonstrated that CSCs secrete higher levels of angiogenic factors and exhibit superior angiogenic function compared to those of ACSs.

### 2.8. ACSs Show Strong Anti-Inflammatory Function

It is well known that both the amnion and chorion are composed of high-level inflammatory factors. Here, we performed a direct comparison with regard to their anti-inflammatory functions by using conditioned media from cultured CSCs and ASCs. We first cocultured a monocyte cell line, THP-1, with supernatant from either CSCs or ASCs. After stimulation with Lipopolysaccharide (LPS), we examined the levels of cytokine, TNF-a, as an indicator of inflammation. We found significantly decreased TNF-a secretion in both the CSC and ASC samples (Figure 4C). However, when we determined the level of TSG-6, an anti-inflammatory factor, the results showed that the secretion of TSG-6 in the supernatant from ASCs is two-fold higher than the supernatant from CSCs (Figure 4D). TSG-6 is considered an essential anti-inflammatory factor in the context of wounds and other inflammatory environments. Here, we showed that ASCs secreted higher levels of TSG-6 than CSCs.

## 3. Discussion

The human placenta contains non-immunogenic cells, growth factors, cytokines, and an extracellular matrix (ECM), making it a potent solution for a variety of indications due to its angiogenic, anti-inflammatory, anti-oxidative, anti-microbial, and anti-fibrotic properties. The MSCs, rich extracellular matrix, key cytokines, and growth factors in the tissues contribute to the functions of these properties. It is believed that the extracellular matrix, cytokines, and growth factors are mainly from the secretions of the MSCs within the tissues. Therefore, the MSCs residing in the tissues at least partially dominate the functional properties of the amnion membrane, chorion membrane, and umbilical cord. MSCs have been shown to have regenerative functions, including angiogenic, anti-inflammatory, anti-oxidative, anti-microbial, and anti-fibrotic properties. MSCs can be isolated from all layers of a full-term placenta, including from the amniotic membrane, chorionic membrane, chorionic villi, decidua, and umbilical cord. Comparing MSCs from different parts of the placenta will facilitate the selection of appropriate MSCs for clinical applications. Several studies have isolated and investigated the characteristics of these cells after in vitro culture. However, a comparison of the freshly isolated cells before putting them in culture has not been reported [36]. Here, for the first time, we systematically characterized the gene expression profiles of directly isolated human amniotic stromal cells and human chorionic stromal cells from term pregnancies.

We identified differentially expressed genes, including the highly expressed genes in ASCs, *LMO3* and *UTY*, and the highly expressed genes in CSCs, *NAA11, BIRC7, APOD, HOXA11, HOXA13, MKRN4P, RGPD1,* and *NR2F1*. Among these genes, *LMO3* is a member of the LIM-domain-only (LMO) protein family. Studies have reported that *LMO3* is involved in the transcriptional regulation of specific target genes in collaboration with other transcription factors, such as p53, as well as the regulation of cell invasion and proliferation [37,38,39]. *UTY* is the human ubiquitously transcribed tetratricopeptide repeat gene, and it encodes histone demethylase, which is involved in protein–protein interactions, cell proliferation, and differentiation [40]. Evidence of the UTY protein at the protein level predicted intracellular and secreted proteins. Interestingly, both the *LMO3* and *UTY* genes are related to gene epigenetic regulations, and their high expression in ACSs but not in CSCs suggests that distinct machineries of epigenetic regulation exist in ACSs and CSCs. *NAA11* expression was detected only in the testis and placenta obtained from normal human subjects [41]. Here, we showed that *NAA11* is only expressed in CSCs; however, no detectable expression was found in ASCs. Therefore, *NAA11* may serve as a biomarker for identifying the origin of CSCs from ASCs. It has been shown that *Hoxa11* and *Hoxa13* suppression result in a significant reduction of Sox9 and collagen type 2 expression, suggesting a reduction in chondrogenic potential [42,43,44]. High expression of *HOXA11* and *HOXA13* in CSCs demonstrates that these cells have higher chondrogenic potential than ASCs. It is important to note that during our data analysis, long non-coding RNA (lncRNA) identification and annotation were at a preliminary stage [45,46]. Therefore, further investigation is necessary in order to identify potential novel lncRNAs.

Alternative splicing is an important posttranscriptional process that enables a single gene to produce multiple distinct transcripts, namely isoforms. These isoforms carry different biological properties that are different in catalytic ability, subcellular localization, or protein interaction. We identified the 10 most significantly differentially expressed isoforms, including both known and novel isoforms. These isoforms included *CXCL6, FOXF1-AS1, HGF, MAEL, NPTX1, HGF, ATP2A3, DPT, POSTN*, and a novel isoform (Isoform ID: XLOC_040015). Among them, *HGF, MAEL, NPTX1, ATP2A3,* and *DPT* were highly expressed in CSCs, and *CXCL6, FOXF1-AS1, POSTN,* and the novel isoform (Isoform ID: XLOC_040015) were highly expressed in ASCs. The naturally occurring isoforms of HGF, NK1, and NK2 are expressed in human tissues during development and in normal adults [47]. Studies in cell culture and in transgenic animals have suggested that NK1 is capable of recapitulating normal HGF signaling and biological activities, while NK2 appears to be an antagonist for HGF-induced cellular proliferation. NK2 expression is increased relative to full-length HGF in human fibrotic organ diseases, and it is possible that NK2 may play a role in the failure of normal repair [47]. The normal biological roles of the truncated HGF isoforms remain to be determined. Further understanding of the normal functions of these isoforms, similarly to the novel isoforms identified in this study, may provide insight into the characteristic properties of CSCs and ASCs.

Our GO analysis showed that the GO terms of extracellular matrix, angiogenesis, and cell adhesion were significantly enriched in CSCs. We further explored the angiogenic function of CSCs due to its importance in regenerative medicine. Increased levels of angiogenin, VEGFA, HGF, and bFGF protein and enhanced tube formation in vitro proved that CSCs exhibited a strong angiogenic function. Although inflammation did not fall into the top 10 GO terms, we realized that many key genes and/or their isoforms (Table 1, Table 2 and Table 3) that were involved in inflammation were significantly expressed between ASCs and CSCs, such as CXCL6, IL-1B, HOXA11, HOXA13, HGF, CSF1, and NR2F1, suggesting that the different regulation machineries for the secretion of anti-inflammatory factors exist in these two populations. Therefore, we investigated the anti-inflammatory response in this study in terms of inhibition of an inflammatory factor, TNF-a, and enhancement of secretion of an anti-inflammatory factor, TSG-6. Interestingly, ASCs induced two-fold higher TSG-6 secretion than CSCs. Furthermore, among the GO terms that were found to be different, there were also negative regulators of cell proliferation, although our preliminary observation did not show different proliferation rates between ASCs and CSCs. Further investigation is warranted in order to confirm these observations due to the importance of the physiology of these cells, such as in cell proliferation, senescence, and apoptosis.

It is important to note that freshly isolated heterogenous cell populations were used in this study. Further in vitro and in vivo experiments with phenotypic and morphologic characteristics, as well as differential capacities, are warranted in order to characterize the MSCs isolated from amnions and chorions. It will be of special interest to describe the differentiation and progenitor potential of these amnion and chorion MSCs, as well as the full spectrum of secreted factors.

Mesenchymal stromal cells derived from the fetal placenta have emerged as promising resources for regenerative medicine. Our study demonstrated that CSCs have superior angiogenic potential, but ASCs exhibit increased anti-inflammatory properties. Therefore, both CSCs and ASCs could potentially be the sources of MSCs for regenerative medicine based on the required functional properties. The current results add to the rapidly expanding field of interest in the potential research and therapeutic applications of the MSCs derived from fetal placenta membranes.

## 4. Materials and Methods

### 4.1. Tissue Procurement and Ethics Statement

Placental tissue with procurement and ethics statements that were collected from eligible donors after obtaining written consent was purchased from The National Disease Research Interchange (Philadelphia, PA, USA) and Cord Blood America, Inc. (Las Vegas, NV, USA) [48].

### 4.2. Placental Tissue Processing and Isolation of the Amniotic Membrane and the Chorionic Membrane

Placental research tissues were aseptically processed as described previously [49,50]. The amniotic membrane was physically separated from the chorionic membrane. The chorionic membrane (CM) was separated from the decidua via blunt dissection, followed by a wash in anticoagulant citrate dextrose, solution A (ACD-A) (Fenwal Inc., Lake Zurich, IL, USA). The CM was then subjected to enzymatic treatment with dispase solution (1:20 dilution) (Corning Inc, Corning, NY, USA) to allow for separation of the stromal layer of CM from the trophoblast layer, the choriodecidua, and the decidua. The stromal layer of CM was washed with saline and mechanically cleaned to remove residual blood and trophoblasts. The membrane was then incubated for 24–48 h in an antibiotic cocktail solution as described before [48]. The CM was rinsed in Dulbecco’s phosphate buffered saline (DPBS), and then CSCs were isolated through enzymatic membrane digestion.

### 4.3. Isolation of ASCs and CSCs from the Amniotic Membrane and Chorionic Membrane

To isolate CSCs from the chorionic membrane, the membrane was incubated in 1 mg/mL type II collagenase (Worthington Biochemical Cporporation, Lakewood, NJ, USA) at 37 °C, followed by filtering in 100, 70, and 30 μm nylon strainers. The cell digest was centrifuged (Beckman Coulter GS-6R) at 1500 rpm to pellet the CSCs, and a cell count was obtained using a Cellometer (Nexcelom Bioscience, Lawrence, MA, USA). To isolate ASCs from the amniotic membrane, the amniotic membrane was first treated with 0.25% trypsin for 25 min to remove amniotic epithelial cells. After the membrane was washed twice with DPBS, ASC isolation was performed in the same way as the CSC isolation mentioned above. Freshly isolated, uncultured ASCs and CSCs were used for RNA-seq, and cells with passages below five were used for the entire in vitro study.

### 4.4. Cell Culture and Characteristic Analysis through Flow Cytometry

A total 5 × 10^5^ of ASCs and CSCs were cultured in T175 tissue-culture-treated plates containing 30 mL low glucose DMEM medium plus 5% FBS. The culture medium was changed every other day. For the flow cytometry analysis, first-passage ASCs and CSCs were digested with TypLE. After washing with PBS, the cells were resuspended in a cell-staining solution (PBS plus 0.5% Bovine serum albumin (BSA)). The cells were then stained with fluorochrome-conjugated antibodies: IgG isotype-FITC, IgG isotype-PE, IgG isotype-PerCP, IgG isotype-APC, HLA-ABC-FITC, HLA-DR-FITC, CD73-PE, CD45-PE, EpCam-PE, CD146-PerCP, CD90-APC, and CD105-APC (BD Bioscience, San Jose, CA, USA). After 30 min, the stained cells were washed twice with the staining solution. Then, flow cytometry data were acquired by Accuri C6 (BD Bioscience) and analyzed with the FlowJo software (Version 10.7, BD Bioscience, San Jose, CA, USA).

### 4.5. RNA-Seq and Data Analysis

ASCs isolated from three donors and CSCs from four donors were immediately preserved in TRIzol reagent at –80 °C. RNA extraction and RNA-seq were carried out at Qiagen Genomic Services. The library preparation was done using a TruSeq^®^ Stranded mRNA Sample preparation kit (Illumina Inc., San Diego, CA, USA). The starting material (100 ng) of the total RNA was mRNA enriched using the oligo dT bead system. The isolated mRNA was subsequently fragmented using enzymatic fragmentation. Then, first-strand synthesis and second-strand synthesis were performed, and the double-stranded cDNA was purified (AMPure XP, Beckman Coulter, Sykesville, MD, USA). The cDNA was end repaired and 3′ adenylated, Illumina sequencing adaptors were ligated onto the fragments’ ends, and the library was purified (AMPure XP). The mRNA stranded libraries were pre-amplified with polymerase chain reaction (PCR) and purified (AMPure XP). The libraries’ size distribution was validated, and the quality was inspected on a Bioanalyzer 2100 or BioAnalyzer 4200 tapeStation (Agilent Technologies). High-quality libraries were pooled based on equimolar concentrations based on the Bioanalyzer Smear Analysis tool (Agilent Technologies, Wilmington, DE, USA). The library pools were quantified using quantitative PCR (qPCR), and the optimal concentration of the library pool was used to generate the clusters on the surface of a flow cell before sequencing on a NextSeq500 instrument (76 cycles) according to the manufacturer’s instructions (Illumina Inc.). To inspect the mapping in detail, we used the Integrative Genomics Viewer software to browse the BAM files [51,52]. The Tuxedo software package, including Bowtie2 [53], Tophat [54], and Cufflinks [55,56,57], was used for the data analysis. In addition, we performed fragment bias correction, which sought to correct for sequence bias during library preparation [58,59,60]. On average, 64.1 million reads were generated for each sample, and the average genome mapping rate was 96.3%. Only bases with an O-score above 30 were included in the downstream analysis. The mapped reads of the 500 genes that had the largest coefficients of variation based on the fragments per kilobase of transcripts per million (FPKM) [61] were used for principal component analysis (PCA) and heat mapping with unsupervised clustering. Supervised clustering was used to generate a volcano plot and identify differently expressed genes and novel isoforms. Gene Ontology (GO) enrichment analysis for biological processes was used to investigate specific GO terms.

### 4.6. Characterization of Cytokine and Growth Factor Profiles in ASCs and CSCs

ASC and CSC lysates or culture supernatants, which were prepared through an overnight cell culture in a growth medium, were obtained from three donors for the growth factor and cytokine profile determinations. Cells were plated in six-well culture plates (3 × 105 cells per well) in DMEM growth media (supplemented with 10% fetal bovine serum (FBS) and 1% antibiotic/antimycotic). The supernatants were collected and analyzed using a Human Inflammation Panel, 37-Plex (Bio-Rad, Hercules, CA, USA) on a Bio-Plex MAGPIX plate reader (Bio-Rad, Hercules, CA). The test panels included growth factors that are important for anti-inflammation, wound healing, angiogenesis, antimicrobial activity, and osteogenesis: APRIL, BAFF, sCD30, sCD136, chitinase-3-like 1, sIL-6Rβ, IFNα2, IFNβ, IFN-γ, IL-2, sIL-6Rα, IL-8, IL-10, IL-11, IL12 (p40), IL-12 (p70), IL-19, IL-20, IL-22, IL-26, IL-27 (p28), IFNγ2, IFNγ1, IL-32, IL-34, IL-35, LIGHT, MMP-1, MMP-2, MMP-3, osteocalcin, osteopontin, pentraxin, sTNF-R1, sTNF-R2, TSLP, and TWEAK. PGE2, angiogenin, FGF-2, VEGFA, and TSG-6 were analyzed with ELISA (R&D Systems, Menneapolis, MN, USA).

### 4.7. Secretion of Inflammatory Factors by Activated THP-1 cells

The anti-inflammatory capabilities of CSCs and ASCs were characterized with an inhibition assay of the TNF-α produced by LPS-activated THP-1 cells. A total of 500,000 THP-1 cells were plated per well in a 24-well plate, stimulated with 1 ug/mL of LPS in DMEM growth media, and co-cultured with either CSCs or conditioned media from an overnight culture of CSCs and ASCs in growth media. The untreated THP-1 and LPS-treated THP-1 cells represented the positive control and negative control, respectively. The culture was incubated overnight at 37 °C with 5% CO_2_. After overnight stimulation and treatment, the cell media were collected, and the amount of TNF-α produced by the THP-1 cells after LPS treatment was compared to those of the CSC and ASC treatments and reported as percent inhibition vs. the control. TNF-α was quantified with Luminex and/or ELISA (R&D Systems).

### 4.8. HUVEC Tube Formation Induced by ASC and CSC Cell Culture Supernatants

The ASC and CSC culture supernatant dilutions were evaluated in an angiogenesis tube formation assay through stimulation of human umbilical vein endothelial cells (HUVECs; Lonza, Walkersville, MD, USA) to form closed, vessel-like structures. HUVECs were seeded with 1.2 × 10^4^ cells/well on Matrigel (Corning, NY, USA)-coated u-Plate 96-culture wells (Ibidi, Grafelfing, Bayern, Germany). The negative and positive control wells contained endothelial basal medium 2 (EBM-2; Lonza, Walkersville, MD, USA) and endothelial growth medium 2 (EGM-2; Lonza), respectively. The HUVECs were treated with 70 ul of ASC and CSC culture supernatants at 1:4 dilutions in DMEM only, DMEM growth media, and DMEM only. The HUVEC cultures were incubated for 6 h at 37 °C and 5% CO_2_, and images of representative fields were taken at 4× magnification. The magnitude of the tube formation was quantified with the Wimasis software by the number of closed structures.

### 4.9. Statistical Analysis

The results are presented as mean ± standard deviation (SD) for one representative experiment consisting of three donors and two biological replicates. Student’s T-test was used to determine the significance of differences between groups, whereby *p* < 0.05 was considered significant.

## Figures and Tables

**Table 1:** Top 10 most significantly differentially expressed genes.

Gene	CSCs FPKM	ASCs FPKM	Log2 Fold Change	*q*-Value
*NAA11*	3.47	0	−9.82	0.038
*LMO3*	0.02	11.54	9.44	0.009
*UTY*	0.01	4.46	8.93	0.002
*BIRC7*	97.61	0.28	−8.47	0.002
*APOD*	34.72	0.11	−8.28	0.002
*HOXA13*	2.87	0.01	−8.24	0.034
*MKRN4P*	5.69	0.02	−7.98	0.006
*HOXA11*	13.17	0.06	−7.89	0.025
*RGPD1*	0.77	0	−7.87	0.002
*NR2F1*	24.25	0.1	−7.87	0.002

**Table 2:** Top 10 most significantly differentially expressed isoforms.

Gene	CSCs FPKM	ASCs FPKM	Log2 Fold Change	*q*-Value
*CXCL6*	1.28	223.96	7.45	0.021
*XLOC_040015*	0.08	9.7	6.95	0.021
*FPXF1-AS1*	17.86	0.17	−6.71	0.021
*HGF*	308.31	3.13	−6.62	0.021
*MAEL*	17.59	0.2	−6.43	0.021
*NPTX1*	39.61	0.47	−6.39	0.021
*HGF*	1110.15	1.7	−6.02	0.021
*ATP2A3*	34.04	0.75	−5.5	0.021
*DPT*	3.26	136.88	5.39	0.021
*POSTN*	3.39	125.22	5.21	0.021

**Table 3:** Top 10 most significantly differentially expressed novel isoforms.

Closest Known Transcript	CSCs FPKM	ASCs FPKM	Log2 Fold Change	*q*-Value
*HGF*	110.15	1.7	−6.02	0.021
*RP11*	2.11	0.09	−4.52	0.036
*MICAL2*	14.4	0.95	−3.93	0.021
*KRT7*	54.11	7.08	−2.93	0.021
*PERP*	17.19	116.26	2.76	0.021
*ACRC*	0.74	4.95	2.75	0.021
*PLBD1*	14.14	2.39	−2.57	0.021
*IL1B*	52.28	246.72	2.24	0.021
*MEST*	43.64	10.09	−2.11	0.021
*CSF1*	66.89	15.8	−2.08	0.021

**Table 4:** Top 10 most significant GO terms associated with transcripts found to be differentially expressed between ASCs and CSCs.

GO Term	Annoteted	Significant	Exprected	*p*-Value
Extracellular Matrix Organization	322	47	17.75	3.80 × 10^−10^
Angiogenesis	387	57	21.33	4.80 × 10^−10^
Cell Adhesion	1199	151	66.09	1.10 × 10^−9^
Positive Regulation of Transcription	777	76	42.83	3.20 × 10^−9^
Modulation by Virus	336	18	18.52	4.70 × 10^−9^
Negative Regulation of Cell Proliferation	563	62	31.03	2.50 × 10^−8^
Homophilic Cell Adhesion	105	15	5.79	2.80 × 10^−8^
Postive Regulation of Protein Kinase B Signaling	72	13	3.97	1.50 × 10^−7^
SRP-dependent Cotranslational Protein	317	9	17.47	2.10 × 10^−7^
Nuclear-transcribed mRNA Catabolic Process	520	62	28.66	4.00 × 10^−7^

**Table 5:** Key cytokines and growth factors secreted by ASCs and CSCs.

	Conditioned Medium (pg/μg)		Cell Lysate (pg/10^5^ cells)	
Name	ASCs	CSCs	ASCs	CSCs
**IFNα2**	2.26	1.64	0.04	0.02
**IFNβ**	143.02	92.45	2.52	1.54
**IFNγ**	14.85	11.21	0.3	0.18
**IL-2**	22.92	13.66	0.51	0.27
**sIL-6Rα**	1662.59	1202.55	31.29	16.06
**IL-8**	---	670.22	6.02	3.4
**IL-10**	9.6	8.06	0.14	0.11
**IL-11**	---	180.26	1.29	0.18
**IL-12 (p40)**	149.74	104.04	2.51	1.17
**IL-19**	14.23	11.22	1.11	0.6
**IL-20**	9.49	5.72	0.15	0.09
**IL-22**	50.76	20.73	0.32	0.18
**IL-26**	478.9	295.39	8.41	6.07
**IL-27 (p28)**	102.45	65.27	2.03	1.13
**IL-28A (IFNγ2)**	4.87	2.69	0.07	0.03
**IL-29/IFNγ1)**	50.11	31.01	1.25	0.61
**IL-32**	125.38	132.85	1.89	1.32
**IL-34**	299.74	145.86	5.98	3.34
**IL-35**	256.36	181.68	2.68	0.94
**LIGHT/TNFSF14**	22.25	8.97	0.15	0.03
**MMP-1**	67.65	56.42	0.97	0.63
**MMP-2**	731.38	443.49	46.75	30.95
**MMP-3**	926.57	674.78	17.08	11.39
**sTNF-R1**	25.78	33.69	0.41	0.36
**sTNF-R2**	49.91	32.98	0.74	0.55
**TSLP**	13.16	10.46	0.24	0.15
**TWEAK/TNFSF12**	3.6	2.07	0.08	0.05
**PGE2**	252.84	239.12	--	--
**Angiogenin**	--	30.51	--	--
**FGF-2**	--	98.91	--	--
**VEGFA**	--	98.91	--	--

**Table 6:** Relative level of cytokine and growth factors in amnion and chorion tissues.

Protein Name	Amnion Tissue	Chorion Tissue
IL-1RA	****	****
IL-4	*	**
IL-10	*	**
bFGF	***	****
Angiogenin	***	***
Angiopoietin-1	**	**
Angiopoietin-2	**	***
EGF	**	**
HGF	****	****
SDF-1a	**	***
IGFBP-1	****	*****
PDGF-AA	*	**
PDGF-BB	**	***
PIGF	*	*
VEGF-A	*	***
VEGF-D	*	**
TIMP-1	****	****
Periostin	***	***

Note: * >0 pg/μg, ** >100 pg/μg, *** >1000 pg/μg, **** >5000 pg/μg, ***** >1 ng/μg tissues.

## Data Availability

The data presented in this study are available in the article.
